# Systematic analysis of circRNA biomarkers for diagnosis, prognosis and therapy in colorectal cancer

**DOI:** 10.3389/fgene.2022.938672

**Published:** 2022-10-12

**Authors:** Yafei Xiao, Mengyuan Qiu, Cong Tan, Wanting Huang, Shaowen Hu, Xiaowei Jiang, Mingjie Guo, Congcong Wang, Jingyu Liang, Yimei Wu, Mengmeng Li, Quanying Li, Changjiang Qin

**Affiliations:** ^1^ Department of General Surgery, Huaihe Hospital of Henan University, Kaifeng, China; ^2^ Department of Neurology, Peking University People’s Hospital, Peking University School of Medicine, Beijing, China; ^3^ Institute of Biomedical Informatics, Cell Signal Transduction Laboratory, Bioinformatics Center, Henan Provincial Engineering Center for Tumor Molecular Medicine, School of Basic Medical Sciences, Henan University, Kaifeng, China; ^4^ Department of Gynecology and Obstetrics, The First Affiliated Hospital, Zhejiang University School of Medicine, Hangzhou, China; ^5^ Department of Pediatric Orthopaedics, The Third Affiliated Hospital of Zhengzhou University, Zhengzhou, China; ^6^ Department of Thoracic Surgery, The First Affiliated Hospital of Henan University, Kaifeng, China

**Keywords:** biomarkers, circRNA, colorectal cancer, miRNA, ceRNA, exosome

## Abstract

As the third most common cancer and the second leading cause of cancer death worldwide, colorectal cancer (CRC) poses a serious threat to people’s health. In recent years, circRNA has been widely reported as a new biomarker in CRC, but a comprehensive summary and analysis is lacking. This study aims to evaluate the diagnostic, therapeutic and prognostic significance of circRNAs in CRC by systematically analysing their expression patterns, biological functions and clinical significance in CRC. The literature on circRNA in CRC was searched in the PubMed database and included for analysis after screening according to strict inclusion and exclusion criteria. The UALCAN online tool was used to obtain host gene expression data. The miRTargetLink 2.0 was used to predict target genes for miRNAs action in CRC patients. Cytoscape was used to construct circRNA-miRNA-mRNA interaction networks. From the 236 included papers, we identified 217 circRNAs and their associated 108 host genes and 145 miRNAs. Among the 145 miRNAs, 27 miRNAs had no corresponding target genes. After prediction of target genes and differential analysis, a total of 25 target genes were obtained and a circRNA-miRNA-mRNA interaction network was constructed. Among the 217 circRNAs, 74 were associated with diagnosis, 160 with treatment and 51 with prognosis. And 154 of them function as oncogenes while 58 as tumour suppressor genes. In addition, these circRNAs include 32 exosomal circRNAs, which have unique advantages as biomarkers. In total, we summarize and analyze the expression patterns, biological functions and clinical significance of circRNAs in CRC. In addition, we constructed some new circRNA-miRNA-mRNA regulatory axes based on the miRNAs sponged by circRNAs.

## 1 Introduction

Colorectal cancer, as the third most common cancer and the second leading cause of cancer death worldwide, is a serious threat to people’s life and health with a total of 1,931,590 cases of CRC diagnosed and 935,173 deaths worldwide in 2021 (Siegel et al., 2021). Although the overall 5-year survival rate has been improving over the past few decades, the survival rate for patients with advanced CRC is only about 20%, compared with up to 90% for patients with early-stage CRC (Zeng et al., 2018; Shen et al., 2020). Unfortunately, about 56% of CRC patients have advanced cancer at the time of diagnosis (Millas et al., 2015; Siegel et al., 2017). At present, the commonly used clinical methods of tumour detection, such as ultrasound, X-ray, CT, MRI, endoscopy and nuclear imaging, can only detect lesions visible to the naked eye. When the asymptomatic lumps gradually grow to a size, that is, perceived by oneself, some of the tumours are already in the middle or late stages, and some have already metastasized, thus numerous patients have lost the best treatment period. Tumour marker is considered to be one of the best methods for the early detection of asymptomatic microfocal tumours.

Non-coding RNAs (ncRNAs) are a class of endogenous RNAs that are involved in the regulation of gene expression. In recent years, the regulatory role of ncRNAs in a variety of pathophysiological processes has received extensive attention (Anastasiadou et al., 2018). Circular RNA (circRNA) is a novel endogenous non-coding RNA (ncRNA) molecule that can be reversely spliced to produce a circular structure (Liu et al., 2017a). It can regulate gene expression at the transcriptional or post-transcriptional level by sponging at acting as microRNA (miRNA) and are involved in regulating many important biological processes (Cortes-Lopez and Miura, 2016). circRNA is a closed RNA molecule without 5′ cap structure and 3′ poly tail. Circular structure makes it highly resistant to RNA exonucleases and highly conserved evolutionarily (Louis et al., 2019). Similar to the case of exosomes, circRNA was initially considered to be a non-functional product (Sanger et al., 1976). However, with the development of technology, numerous studies have shown that circRNA plays an irreplaceable role in various cancer biological processes. circRNA acts *via* exosome packaging on distant tissues and cells to regulate various signaling mechanisms in tumors (Meng et al., 2017; Liu et al., 2019). Exosomal circRNA has recently received more attention and is considered to be one of the most promising biomarkers for the future.

circRNA is of stability, specificity, universality and conservation and may be used as a tumour marker and potential therapeutic target in clinical applications (Jeck et al., 2013; Zhou et al., 2018). Although there are many related studies on circRNAs in CRC, these studies are scattered and lack systematic organization and summary. Therefore, this study summarizes and analyzes the expression patterns, biological functions and clinical significance of circRNAs (including exosomal circular RNAs) and their corresponding miRNAs, host genes and target genes in CRC. In addition, given that some miRNAs in the circRNA regulatory axis do not have corresponding target genes, we made some predictions based on the corresponding software and organized them into circRNA-miRNA-mRNA regulatory axis. To our knowledge, this article is the first in CRC field to summarize and systematically analyze circRNA as diagnostic, therapeutic and prognostic markers in such a comprehensive manner, which we hope will shed a light on future research.

## 2 Materials and methods

### 2.1 Screening the literatures and looking for circRNAs

**FIGURE 1:**
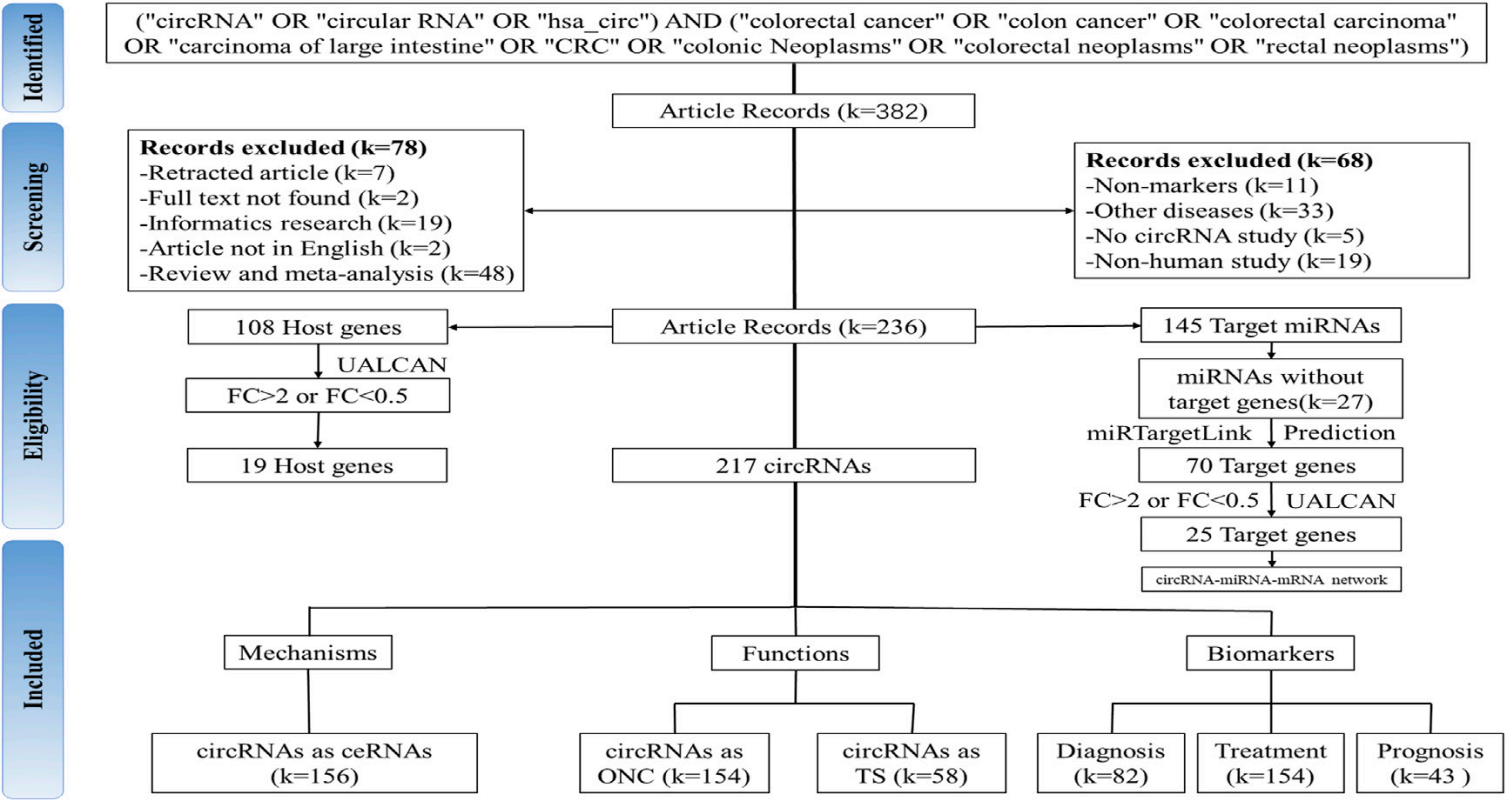
Flow-chart diagram of this study. (k: number of records. ONC: oncogenes. TS: tumour suppressor gene).

The PubMed database, the most commonly used information resource in the biomedical field, provides comprehensive searches on specific topics and is free and easy to use (Falagas et al., 2008). To screen circRNAs as diagnostic, therapeutic and prognostic markers for CRC, we searched the PubMed database (https://pubmed.ncbi.nlm.nih.gov/) on 4 December 2021. Search strategy: (“circRNA” OR “circular RNA” OR “has_circ”) AND (“colorectal cancer” OR “colon cancer” OR “colorectal carcinoma” OR “carcinoma of large intestine " OR “CRC” OR “colonic neoplasms” OR “colorectal neoplasms” OR “rectal neoplasms”). Eligible studies should meet the following criteria: 1) patients with a gold standard definitive diagnosis of CRC; 2) independent original studies assessing differential circRNA expression in CRC tissue; 3) as a diagnostic, therapeutic or prognostic marker for CRC. Exclusion criteria were as follows: 1) reviews, meta-analyses and pure bioinformatics; 2) case reports; 3) no clinical samples; 4) no circRNA studies; 5) non-human studies: cell lines or mice. The screened circRNAs were then categorized and summarized according to their roles in CRC. Besides, we categorized circRNAs as therapeutic markers that were not clearly indicated as CRC biomarkers but were associated with cancer proliferation, invasive metastasis, and progression. Two authors (Y.X. and S.H.) independently extracted data from the included studies using a standardized table that included the following items: name of circRNA, expression (up- or down-regulated), host gene, target gene, sponged miRNA, sample size, study method, mechanism, regulate pathways, in CRC, function, prognosis, biomarker type. All assessments were conducted independently by two investigators to ensure accurate study inclusion. The checklist of circRNA-related literatures and results of assessment are shown in Supplementary Table S1. The flow chart of this study is shown in Figure 1.

### 2.2 Expression of host genes in colorectal cancer

All expression data of host genes were obtained from the TCGA database. The UALCAN online tool is one of the tools that enables in-depth analysis of TCGA data (Chandrashekar et al., 2017). UALCAN is open and free to use at http://ualcan.path.uab.edu.

### 2.3 Prediction of miRNAs’ target genes and construction of circRNA-miRNA-mRNA network

The miRTargetLink 2.0 was used to predict target genes for the action of miRNAs in human CRC patients and is available for free at https://www.ccb.uni-saarland.de/mirtargetlink2 (Kern et al., 2021). Cytoscape (https://cytoscape.org/) is a free JAVA-based tool capable of mapping biomolecular interaction networks (Shannon et al., 2003). circRNA-miRNA-mRNA interaction network maps are constructed by Cytoscape software.

## 3 Results

From the initial search of 382 literatures, 236 literatures were finally included after removing 146 ineligible literatures according to the exclusion criteria. We identified 217 circRNAs biomarkers associated with CRC from these and analyzed their potential role in diagnosis, treatment and prognosis. By removing duplicates, 108 host genes and 145 miRNAs were screened (Supplementary Table S2). Moreover, we analyzed the expression patterns of these host genes and predicted target genes of miRNAs sponged by circRNAs. Finally, a circRNA-miRNA-mRNA interaction network was constructed based on the predicted target genes. Notably, because of the special existence of the exosomal circRNA, we have analysed it separately.

### 3.1 Expression level of host genes in colorectal cancer

circRNAs are steady-state byproducts of partial fragment splicing of host genes (Kristensen et al., 2019). A total of 108 host genes were collected, and 92 genes were available in the TCGA database on the UALCAN platform for expression profiles (Supplementary Table S3). Significant differences with *p* < 0.05 in expression between normal and cancerous tissues were found for 78 genes. Among them, two host genes were down-regulated (FMN2 and NOX4) and the remaining 76 genes were up-regulated. What’s more, the expression levels of 19 host genes differed more than 2-fold change between tumour and normal tissues, 17 host genes of which were up-regulated and two host genes of which were down-regulated (Table 1, Supplementary Table S3). Although circRNAs are derived from the host gene (Lv et al., 2018), we did not observe a clear relationship between circRNAs and their host genes in terms of expression patterns.

**TABLE 1:** Description of circRNAs in colorectal cancer (Host genes expression with *p* < 0.05 and >2-fold change (fold-change >2 or <0.5) in cancer tissues compared with normal tissues.).

circRNA	Exp	Oncogene/TS	Host gene	Exp	Sponged miRNA	Target gene	Function	Biomarker	PMID
circ_0005100	Up	Oncogene	FMN2	Down	miR-1182	hTERT	-	Diagnosis, treatment, prognosis	31738400
circFMN2	Up	Oncogene	FMN2	Down	-	-	-	Diagnosis, prognosis	33337836
circDENND4C	Up	Oncogene	DENND4C	Up	miR-760	GLUT1	Proliferation, migration	Treatment	32196590
circFNDC3B	Down	TS	FNDC3B	Up	-	-	Proliferation, invasion	Prognosis	32241279
circLONP2	Up	Oncogene	LONP2	Up	miR-17-5p	-	Invasion, migration	Prognosis	32188489
circ_0019230	Down	TS	PLCE1	Up	-	-	Proliferation, migration, invasion, apoptosis	Treatment	34173324
circPLCE1	Down	TS	PLCE1	Up	-	-	Proliferation, metastasis	Prognosis	34412652
circ_0059354	Up	Oncogene	RASSF2	Up	miR-195-5p	FZD4	Proliferation, migration, invasion, apoptosis	Treatment	33929991
circ_0026782	Down	TS	ITGA7	Up	miR-370-3p	NF1	Proliferation, metastasis,proliferation,migration	Diagnosis, treatment	29943828
circITGA7	Down	TS	ITGA7	Up	miR-3187-3p	ASXL1	Proliferation	Diagnosis, treatment	31372051
circ_0004680	Up	Oncogene	CCT3	Up	miR-613	WNT3/VEGFA	Invasion	Treatment	31859543
circABCC1	Down	TS	ABCC1	Up	-	-	-	Diagnosis	31669510
circPRKDC	Up	Oncogene	PRKDC	Up	miR-198	DDR1	Proliferation, migration, invasion	Treatment	33364834
circSLC7A6	Up	Oncogene	SLC7A6	Up	-	-	Proliferation, invasion	Prognosis	32423804
circSTIL	down	TS	STIL	Up	-	-	-	Diagnosis	31669510
circ_0030632	Up	Oncogene	UGGT2	Up	-	-	-	Treatment	32115780
circSPARC	Up	Oncogene	SPARC	Up	miR-485-3p	JAK2	Migration, proliferation	Diagnosis, prognosis	34074294
circ_0060745	Down	TS	cse1l	Up	miR-eIF4A3	PCNA	Proliferation	Treatment	32857753
circ_0004887	Up	Oncogene	AURKA	Up	-	-		Treatment	32115780
circPVT1	Up	Oncogene	PVT1	Up	miR-145	-	Proliferation, migration, invasion	Treatment	30922567
circNOX4	Up	Oncogene	NOX4	Down	miR-485-5p	CKS1B	Proliferation, migration, invasion	Treatment	32901890
circKIAA1199	Up	Oncogene	KIAA1199	Up	miR-34c-5p	MSI1	Proliferation, migration, invasion	Diagnosis	34387591

### 3.2 Regulatory mechanism of circRNA in colorectal cancer

**FIGURE 2:**
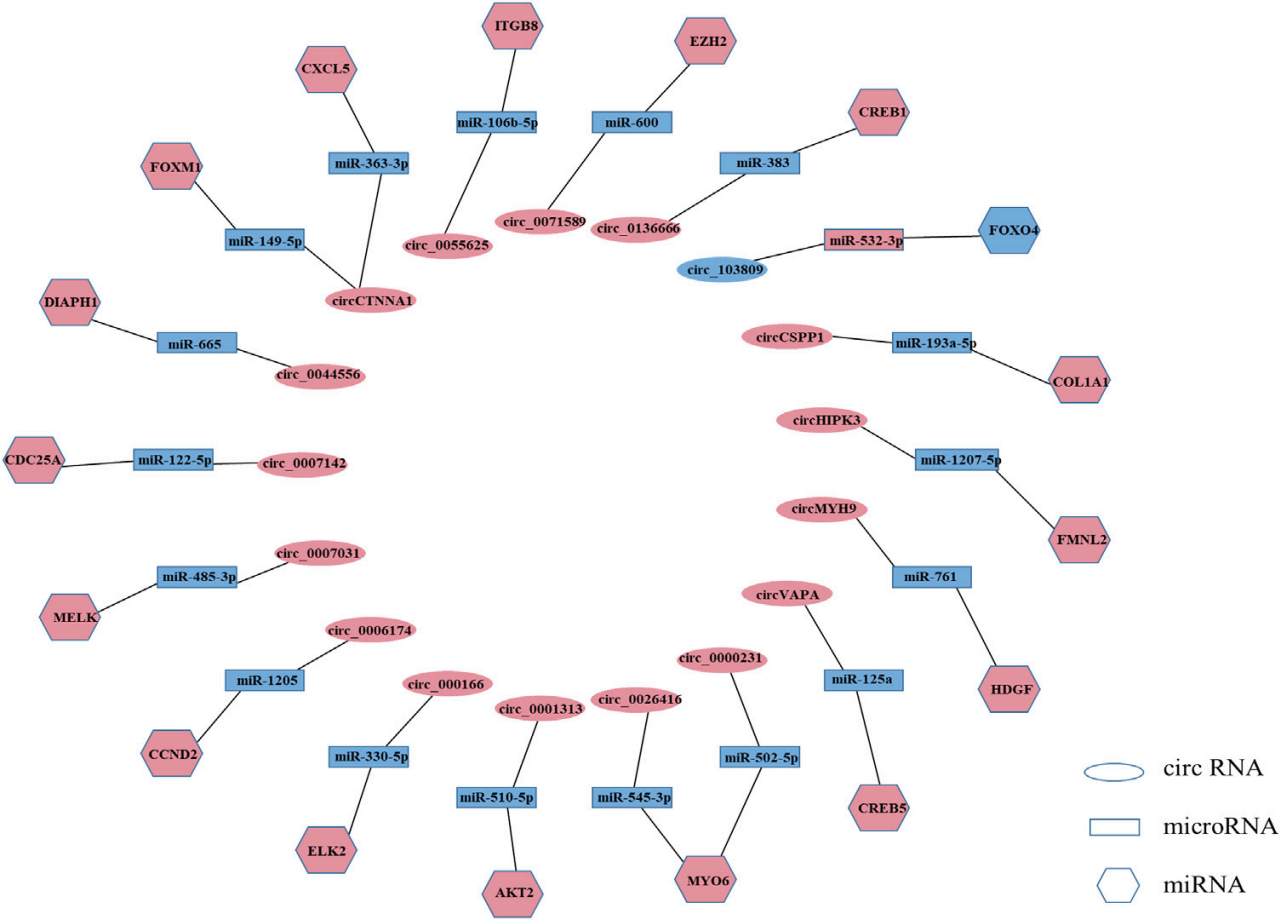
Regulatory mechanisms of circRNAs in colorectal cancer. (Light coral represents high expression, and blue represents low expression. Only circRNAs that were reported repeatedly are listed in this figure).

MicroRNAs (miRNAs) are a class of non-coding RNAs consisting of approximately 22 nucleotides that bind to the 3ʹ-untranslated region (3ʹUTR) of their target genes to induce or repress the translational expression process (Zhang et al., 2017a). circRNA is a novel non-coding RNA with a covalently closed continuous loop, which makes it more stable than linear microRNAs with 3′ and 5′ ends (Nigro et al., 1991; Jeck et al., 2013; Memczak et al., 2013; Chen and Yang, 2015). circRNA can act as miRNA sponge and regulate gene expression (Zhang et al., 2017b; Panda, 2018; Chen et al., 2020a). As miRNA sponges, circRNAs are most notable for their competitive endogenous RNA (ceRNA) role (Zhang et al., 2019a). In the ceRNA network, circRNAs can bind to miRNAs through their miRNA binding sites (also known as miRNA response elements [MREs]), thereby regulating the mRNAs of the corresponding miRNAs’ target genes and attenuating the repressive effects of miRNAs on target genes (Hansen et al., 2013; Memczak et al., 2013; Xi et al., 2021). circRNA-miRNA-mRNA network plays a key role in cancer and non-cancer pathways (Jin et al., 2016; Yang et al., 2019), such as bladder cancer (Huang et al., 2016), colorectal cancers (Weng et al., 2017), osteosarcoma (Liu et al., 2017b), and human cartilage degeneration (Liu et al., 2016). Among the circRNAs we collected, more than a quarter of circRNAs exerted tumor suppressor or tumor-promoting effects in colorectal cancer through the ceRNA mechanism. For example, circVAPA can act as sponge of miR-125a to suppress colorectal cancer cell growth process by regulating miR-125a/CREB5 axisplay (Zhang et al., 2020a). circ_103809 can exert tumor suppressor effect through miR-532-3p/FOXO4 axis (Bian et al., 2018) (Supplementary Table S2, Figure 2).

### 3.3 Prediction of miRNAs’ target genes and construction of circRNA-miRNA-mRNA network

**FIGURE 3:**
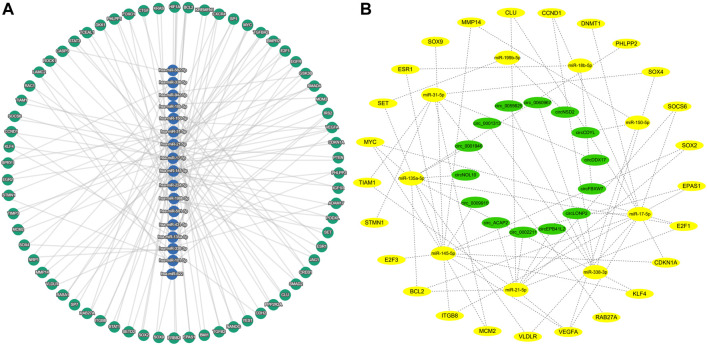
Predicted target genes and construction of circRNA-miRNA-mRNA network **(A)** miRNA and predicted target genes regulated by at least two miRNAs. **(B)** circRNA-miRNA-mRNA network in colorectal cancer.

Some circRNAs have corresponding miRNAs, but lack the corresponding target genes. Among the 145 miRNAs, 27 miRNAs have no responsive target genes. The predicted target genes of these miRNAs were conducted on the miRTargetLink 2.0 online platform, and finally 3567 target genes were obtained. After excluding the weakly correlated target genes, only 496 target genes were left. Among these target genes, 70 target genes regulated by at least two miRNAs were identified (Figure 3A, Supplementary Table S4).

70 target genes were analyzed in the UALCAN platform, and 25 target genes were finally obtained by eliminating the ineligible genes (*p* > 0.05 and 0.5 < Fold-change <2) (Supplementary Table S5). The miRNAs corresponding to these 25 target genes were identified in Supplementary Table S4, and the corresponding circRNAs were then found in the Supplementary Table S2 according to these miRNAs. Based on the circRNA-miRNA-mRNA correspondence, the network map was generated in the Cytoscape software (Figure 3B).

### 3.4 circRNA’s functions in colorectal cancer

**FIGURE 4:**
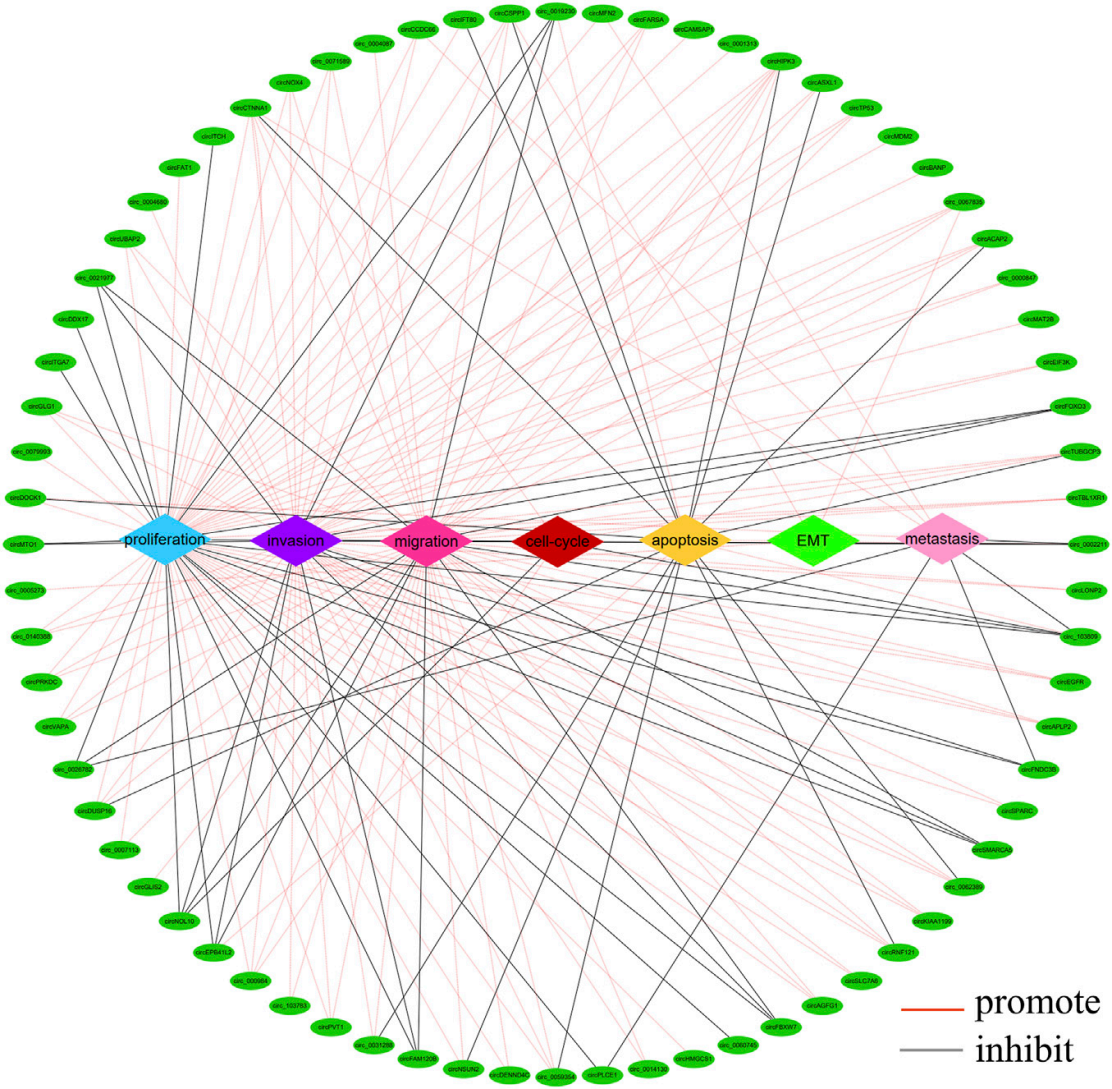
Network of aberrantly expressed circRNAs in diverse cellular functions. (The figure shows only circRNAs with a *p* < 0.05 in host gene expression).

There is growing evidence that circRNAs play an important role in the development of CRC as oncogenes or tumour suppressor genes. circRNAs are involved in different processes of tumour pathogenesis, including cell proliferation, migration, invasion, apoptosis, metastasis, epithelial mesenchymal transition (EMT) and cell cycle (Yin et al., 2020; Zhang et al., 2021a; Su et al., 2022). A summary analysis of the literature revealed that most circRNAs were associated with cancer cell proliferation (*n* = 64), invasion (*n* = 48), migration (*n* = 49) and apoptosis (*n* = 18), while a few cyclic RNAs were involved in cancer cell metastasis (*n* = 9), cell cycle (*n* = 5) and EMT (*n* = 3) (Figure 4). Of the 217 circRNAs we studied, 154 functioned as oncogenes, 58 tumour suppressor genes and five controversial genes (some publications reported them as oncogenes, while others reported them as tumour suppressors), which are circ_0000338, circ_0060745, circ_103,809, circCCDC66, circZNF609.

#### 3.4.1 Function as oncogenes

Some circRNAs are up-expressed in CRC tissues and cells, and play an oncogenic role by regulating downstream target genes or activating certain signaling pathways. In 154 oncogenes, 23 circRNAs were repeatedly studied, among which the most frequently studied were circ_0007142 (Zhu et al., 2019; Yin et al., 2020; Wen et al., 2021) and circCSPP1 (Wang et al., 2020a; Li et al., 2021; Xi et al., 2021).

circ_0007142 was found to be significantly upregulated in CRC and is associated with poor differentiation and lymphatic metastasis. Overexpressed circ_0007142 promotes proliferation, migration, and invasion of CRC cells by targeting miR-103a-2-5p/PARP-1, and subsequently activating Wnt/β-Catenin pathway (Zhu et al., 2019). Another article indicated that circ_0007142 also regulated miR-455-5p/SGK1 to inhibit cell apoptosis of CRC (Yin et al., 2020). In addition, Yin et al. revealed the third way for circ_0007142 to play an oncogenic role through miR-122-5p/CDC25A (Wen et al., 2021).

A study has demonstrated that the upregulation of circCSPP1 in CRC tissues and cells promoted cell proliferation, migration, invasion, and inhibited apoptosis by regulating miR-431/LASP1 axis (Li et al., 2021). Moreover, activation of the circCSPP1/miR-193a-5p/COL1A1 facilitated EMT and liver metastasis (Wang et al., 2020a). Xi et al. (2021) confirmed the conclusion in Doxorubicin-resistant CRC cells, and found upregulated circCSPP1 reduced doxorubicin sensitivity through miR-944/FZD7 axis.

#### 3.4.2 Function as tumour suppressor genes

In addition to oncogenes, 58 circRNAs are down-regulated in CRC and have a suppressive effect on tumour proliferation, invasion and migration. Two of them have been repeatedly reported, which are circFBXW758 (Lu et al., 2019; Xu et al., 2021) and circFNDC3B (Pan et al., 2020; Zeng et al., 2020).

circFBXW7 is reduced in CRC patients and exerts cancer-inhibiting effects, suppressing cell proliferation, migration and invasion in CRC. Mechanically, low expression of circFBXW7 prohibited the progression of CRC through NEK2, mTOR, and PTEN pathways (Lu et al., 2019).To understand the effect of circFBXW7 on chemoresistance, Xu et al. (2021) transferred circFBXW7 into oxaliplatin-resistant CRC cells. They found that circFBXW7 increased oxaliplatin-induced apoptosis and inhibited oxaliplatin-induced EMT, increasing the sensitivity of drug-resistant cells to oxaliplatin.

It has been shown that circFNDC3B is downregulated in CRC patients who have a poor prognosis with shorter OS than patients with overexpressed circFNDC3B. circFNDC3B could encode a novel protein circFNDC3B-218aa to inhibit the proliferation, invasion and migration of CRC cells by reducing the expression of Snail (Pan et al., 2020). Besides, circFNDC3B could also modulate CRC growth, angiogenesis and liver metastasis by circFNDC3B/miR-97-5p/TIMP3 axis (Zeng et al., 2020). These studies suggested that circFNDC3B may be a potential prognosis and therapeutic biomarker for CRC.

**FIGURE 5:**
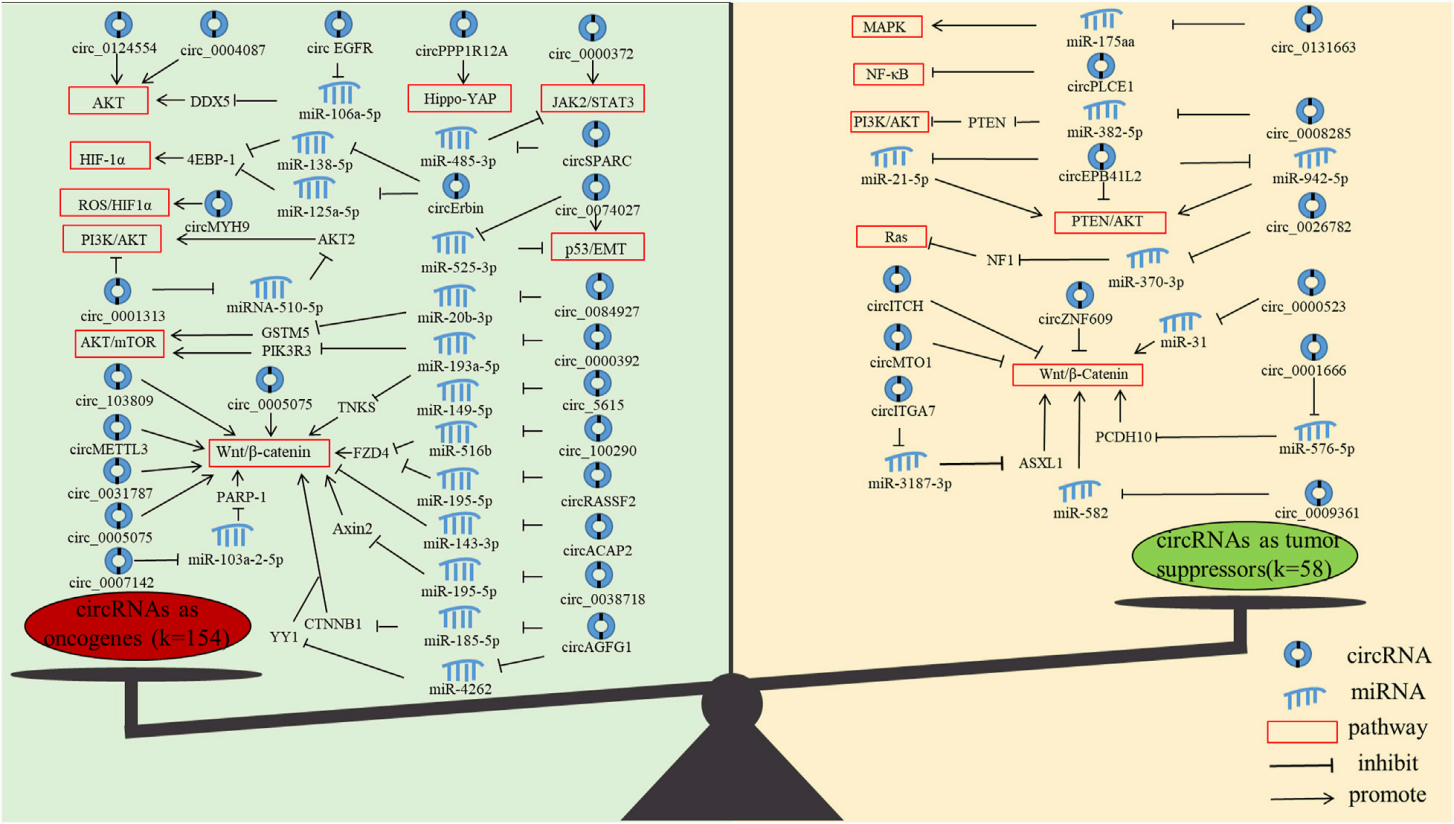
The molecular functions of circRNAs in CRC progression. (The figure shows only circRNAs with signaling pathways.)

In addition, we found that the current research on circRNAs mainly focused on oncogenes, and less research is performed on tumour suppressor genes. Oncogenes mainly function through AKT, JAK2/STAT3, AKT/mTOR, HIF-1α, Hippo-YAP, NF-κB, p53/EMT, PI3K/AKT, ROS and Wnt/β-Catenin pathways, and tumor suppressor genes mainly function through MAPK, NF-κB, PI3K/AKT, PTEN/AKT, Ras and Wnt/β-Catenin pathways (Figure 5). Notably, the most studied pathway for both oncogenes and tumour suppressor genes is the Wnt/β-Catenin pathway. The Wnt/β-Catenin pathway plays an integral role in embryogenesis and adult homeostasis *in vivo* (Nusse and Clevers, 2017). Aberrant activation of this pathway is associated with growth-related diseases and cancers, particularly as a key promoter of CRC development and progression (Vallee and Lecarpentier, 2018; Bian et al., 2020).

### 3.5 The role of circRNA in the diagnosis, treatment and prognosis of colorectal cancer

Of the 217 circRNAs, 82 were associated with diagnosis, 154 with treatment and 43 with prognosis. Among these circRNAs, 30 circRNAs were repeatedly reported (≥2). Among them, six circRNAs have been reported several times as diagnostic markers (circ_0000338, circ_0001178, circ_0006174, circ_0007142, circ_103,809, circZNF609); 20 circRNAs have been reported repeatedly as treatment markers (circCSPP1, circCCDC66, circZNF609 etc.) and two circRNAs have been reported repeatedly as prognosis markers (circ_0005075, circ_100,876) (Supplementary Table S6). We next focused on those circRNAs reported repeatedly.

#### 3.5.1 circRNAs act as the diagnostic biomarkers in tumours

There are six circRNAs (circ_0000338, circ_0001178, circ_0006174, circ_0007142, circ_103,809, circZNF609) that have been repeatedly reported as diagnostic markers. Among them, circ_0001178, circ_0006174, and circ_0007142 were all found overexpressed in CRC, and were considered as diagnostic biomarkers of CRC. In addition, circ_103809 has been shown to be down-expressed in CRC tissues and is considered as a potential novel biomarker for CRC diagnosis.

Both circ_0000338 and circZNF609 were associated with the diagnosis of CRC, but notably, their expression patterns were controversial in different reports. Down-regulation of circ_0000338 in CRC was detected by qRT-PCR by Hon et al. (Hon et al., 2019). However, Zhao et al. found that circ_0000338 expression was significantly higher in CRC tissues than in paracancerous tissue (Zhao et al., 2021). In addition, opposite descriptions of circZNF609 expression in CRC were also described in different papers (Oliveira et al., 1988; Zhang et al., 2019b). Further experiments are needed to verify the expression pattern of these controversial circRNAs in CRC tissues.

#### 3.5.2 circRNAs act as the treatment biomarkers in tumours

Among the 20 circRNAs repeatedly reported as therapeutic markers, circFBXW7 was down-regulated, 17 circRNAs were up-regulated, and two circRNAs (circ_0060745 and circZNF609) had inconsistent expression patterns in different reports. These 17 circRNAs were reported twice, except for circCSPP1, circZNF609 and circCCDC66, which were reported three times in duplicates.

circCSPP1 acts as an oncogene in three literature reports. Wang et al. found that circCSPP1 was significantly up-regulated in CRC tissues, and that circCSPP1 promotes the migration and invasion of colorectal carcinoma cells *in vitro* and *in vivo*. In addition, they found that circCSPP1 may act as a promising therapeutic target by regulating the EMT process in CRC *via* activation of the circCSPP1/miR-193a-5p/COL1A1 axis (Wang et al., 2020a). Xi et al. found that circCSPP1 knockdown inhibited the growth of Doxorubicin-resistant CRC cells and enhanced doxorubicin sensitivity through the miR-944/FZD7 axis, providing a potential target for CRC therapy (Xi et al., 2021). In addition, it was also found that knockdown of circCSPP1 promoted apoptosis and weaken tumour growth *in vivo*, providing evidence for circCSPP1 as a promising biomarker for CRC management (Li et al., 2021).

It has been reported that circCCDC66 facilitates the development of CRC cells under hypoxic conditions *via* regulation of miR-3140/autophagy pathway, and this finding may provide a novel therapeutic option for patients with CRC (Feng et al., 2020). Furthermore, Hsiao et al. found that circCCDC66 controlled multiple pathological processes, including cell proliferation, migration, invasion (Hsiao et al., 2017).

Whether circZNF609 functions either as an oncogene or as a tumour suppressor gene in CRC is controversial. Zhang et al., 2019b found that circZNF609 was down-regulated at the RNA and protein levels. Overexpression of circZNF609 could induce apoptosis, up-regulate the expression of the pro-apoptotic protein Bax, down-regulate the expression of the anti-apoptotic protein Bcl-2, and up-regulate the expression of p53. However, Wu et al. (2018) found that circZNF609 was upregulated at both the RNA level and protein level, regulating the expression of Gli1 through miR-150 and thus promoting CRC migration.

Besides circZNF609 is controversial, the expression of circ_0060745 and circFBXW7 in CRC is also controversial. Some results show that circ_0060745 is up-regulated in CRC and promotes CRC cell proliferation and metastasis by regulating miR-4736/CSE1L signaling pathway, which is considered a novel target for the treatment of CRC (Wang et al., 2020b). However, Zhang et al. found that the expression of circ_0060745 was down-regulated in CRC and inhibited the proliferation of CRC cells (Xu et al., 2020). Further experimental validation is needed to determine their expression pattern in CRC tissues and whether they function as tumour suppressors or oncogenes in humans.

#### 3.5.3 circRNAs act as the prognostic biomarkers in tumours

circ_0005075 and circ_100876 were repeatedly reported as prognostic markers in different literatures. They have been found to be upregulated in CRC and are considered to be potential prognostic markers in CRC. Clinical assays indicated that overexpression of circ_0005075 was significantly associated with histology differentiation, depth of invasion, advanced TNM stage, shorter overall survival and disease-free survival of CRC patients (Jin et al., 2019). Zhong et al. found that circ_0005075 was overexpressed in CRC tissues, and its expression level was correlated with distant metastasis, invasion, tumour lymph node metastasis stage, and tumour diameter of CRC, and was negatively correlated with overall survival of CRC patients (Zhong et al., 2019). Circ_100,876 was reported to be up-regulated in CRC tissues and is closely associated with adverse clinical outcomes (Zhang et al., 2020b; Zhou et al., 2020).

### 3.6 The role of circRNAs in colorectal cancer metastasis and chemoradiotherapy

In addition to circRNAs related to diagnosis, treatment and prognosis, some circRNAs are also correlated with metastasis, drug resistance, and radioresistance. Among the 217 circRNAs, 42 were metastasis-related circRNAs. Among them, 19 were related to lymph node metastasis, 10 were related to distant metastasis, seven were related to both lymph node metastasis and distant metastasis, and the remaining six were related to liver metastasis (Supplementary Table S7). There were 13 circRNAs associated with drug resistance, of which circDDX17 (Ren et al., 2020), circ_0007031 (He et al., 2020), circ_0032833 (Li and Zheng, 2020), circ_0000338 (Zhao et al., 2021), circNRIP1 (Liu et al., 2021) were associated with 5-fluorouracil resistance; circ_0005963 (Wang et al., 2020c), circFBXW7 (Xu et al., 2021) were associated with oxaliplatin resistance; circ_0000338 (Hon et al., 2019), circ_32883 (Abu et al., 2019) were associated with both 5-fluorouracil and oxaliplatin; circ_ 0020095 (Sun et al., 2020), circ_0071589 (Zhang et al., 2021b), and circ_0131,663 (Wang et al., 2021) were associated with cisplatin resistance; circCSPP1 (Xi et al., 2021), and circ_0006174 (Zhang et al., 2022) were associated with Adriamycin resistance. In addition, circ_0001313 was associated with radiotherapy resistance (Wang et al., 2019a) (Supplementary Table S7).

### 3.7 Exosomal circRNA as biomarkers for colorectal cancer

32 exosomal circRNAs were found from 217 circRNAs. Of these, 21 exosomal circRNAs were associated with diagnosis, 13 with treatment and 10 with prognosis. Some exosomal circRNAs have the potential to be multiple biomarkers. For example, circ_0005100 was a diagnostic, therapeutic, and prognostic marker for CRC. circ_0021977 and circ_0067835 are prognostic and therapeutic markers for CRC. circIFT80, circ_0000338 and circEPB41L2 may function as diagnostic markers and therapeutic markers in CRC. In addition, circMBOAT2, circFMN2, circLMNB1, circZNF609 and circ_0007534 have been considered as diagnostic and prognostic markers for CRC. Exosome circRNAs exist in different forms *in vivo*. CircEIF3K, circ_0007334, circRNF121 and circ_0006174 were detected in the cell culture medium, while the other 28 circRNAs were found in blood. Nine exosomes, including circ_0001649, were expressed down-regulated in CRC patients, and another 23 exosomal circRNAs were up-regulated (Table 2).

**TABLE 2:** Basic information on exosomal circRNAs as colorectal cancer biomarkers.

Exosomes	circRNA	Exp	Oncogene/TS	Host gene	Exp	Sponged miRNA	Target gene	Biomarker	PMID
Serum	circ_0001649	Down	TS	-	-	-	-	Diagnosis	29421663
Serum	circ_0000338	Down	TS	FCHSD2	Up	-	-	Diagnosis	31712601
Serum	circ_0004771	Up	Oncogene	-	-	-	-	Diagnosis	31737058
Serum	circ_0005963	Up	Oncogene	ciRS-122	-	miR-122	PKM2	Treatment	31901148
Serum	circ_133	Up	Oncogene	-	-	miR-133a	GEF-H1/RhoA	Treatment	32724467
Serum	circMBOAT2	Up	Oncogene	MBOAT2	Up	miR-519d-3p	TROAP	Diagnosis, prognosis	32796815
Serum	circ_102,049	Up	Oncogene	-	-	miR-761, miR-192-3p	FRAS1	Prognosis	33131207
Serum	circ_0004831	Up	Oncogene	-	-	-	-	Prognosis	33292256
Serum	circFMN2	Up	Oncogene	FMN2	Down	-	-	Diagnosis, prognosis	33337836
Serum	circLMNB1	Up	Oncogene	LMNB1	Up	-	-	Diagnosis, prognosis	33337836
Serum	circZNF609	Up	Oncogene	ZNF609	Up	-	-	Diagnosis, prognosis	33337836
Serum	circIFT80	Up	Oncogene	IFT80	Up	miR-296-5p	MSI1	Diagnosis, treatment	33658855
Serum	circ_0000338	Up	Oncogene	-	-	miR-217, miR-485-3p	-	Diagnosis, treatment	33722958
Serum	circ_001659	Up	Oncogene	-	-	-	-	Diagnosis	33725570
Plasma	circ_0021977	Down	TS	PSMC3	Up	miR-10b-5p	P21/P53	Prognosis, treatment	31595500
Plasma	circ_0067835	Up	Oncogene	IFT80	Up	miR-1236-3p	HOXB7	Prognosis, treatment	31648103
Plasma	circABCC1	Down	TS	ABCC1	Up	-	-	Diagnosis	31669510
Plasma	circCCDC66	Down	TS	CCDC66	-	-	-	Diagnosis	31669510
Plasma	circSTIL	Down	TS	STIL	Up	-	-	Diagnosis	31669510
Plasma	circ_0082182	Up	Oncogene	-	-	-	-	Diagnosis	31700498
Plasma	circ_0000370	Up	Oncogene	-	-	-	-	Diagnosis	31700498
Plasma	circ_0035445	Down	TS	-	-	-	-	Diagnosis	31700498
Plasma	circ_0005100	Up	Oncogene	FMN2	Down	miR-1182	hTERT	Diagnosis, treatment, prognosis	31738400
Plasma	circ_0007534	Up	Oncogene	DDX42	Up	-	-	Diagnosis, prognosis	31938236
Plasma	circFNDC3B	Down	TS	FNDC3B	Up	miR-97-5p	TIMP3	Treatment	32896063
Plasma	circEPB41L2	Down	TS	EPB41L2	Up	miR-942-5p, miR-21-5p	-	Diagnosis, treatment	34022068
Plasma	circCOG2	Up	Oncogene	COG2	Up	miR-1305	-	Treatment	34635639
Plasma	circ_0006282	Up	Oncogene			miR-155	FBXO22	Diagnosis	34860442
Cell culture medium	circEIF3K	Up	Oncogene	EIF3K	Up	miR-214	PD-L1	Treatment	34412616
Cell culture medium	circ_0007334	Up	Oncogene	-	-	miR-577	KLF12	Treatment	34459455
Cell culture medium	circRNF121	Up	Oncogene	RNF121	Up	miR-1224-5p	FOXM1	Treatment	34742305
Cell culture medium	circ_0006174	Up	Oncogene	-	-	miR-1205	CCND2	Diagnosis	34792792

## 4 Discussion

As a novel endogenous non-coding RNA (ncRNA) molecule, circRNA have shown great potential as biomarkers due to their high stability, abundance, evolutionary conservation, and wide distribution in various body fluids and exosomes (Jeck et al., 2013; Zhou et al., 2018). Although previous reviews and meta-analyses have reported some studies of circRNAs in CRC, they are small in size and the analyses are relatively fragmented, lacking a comprehensive systematic summary. Therefore, we performed a systematic analysis of the role of circRNAs as markers in CRC. From the 236 included papers, we identified 217 circRNAs associated with CRC. We comprehensively elucidated the relationship between circRNAs and CRC in terms of molecular mechanisms, functions and clinical applications.

Among the 217 circRNAs, the majority of biomarkers were associated with treatment (*n* = 154), 82 were associated with diagnosis, and 43 were associated with prognosis. Some circRNAs are considered to have multiple marker potential. For example, circ_0005075 is upregulated in CRC and its high expression is related to tumour proliferation, distant metastasis and poor prognosis of patients, and has been reported as three markers for the diagnosis, treatment and prognosis of CRC (Zhong et al., 2019). circEPB41L2 is down-expressed in CRC, promoting tumour cell proliferation, migration, and inhibiting apoptosis. It has also been detected in patient plasma samples, and is considered to be a marker for CRC diagnosis and treatment (Jiang et al., 2021). Notably, many circRNAs play a role in the development of CRC through the ceRNA mechanism, illustrating the importance of the ceRNA mechanism for circRNAs. In addition, the majority of these circRNAs markers are oncogenes, probably due to the relatively low cost of studying oncogenes, resulting in an artificial selection bias.

Relatively, circRNAs that were reported repeatedly may have a high discriminatory significance. Thirty circRNAs were reported repeatedly, of which 23 were up-expressed, two were down-expressed, and five were controversial. Among the 23 highly expressed circRNAs, the most studied were circ_0007142 (Zhu et al., 2019; Yin et al., 2020; Wen et al., 2021) and circCSPP1 (Wang et al., 2020a; Li et al., 2021; Xi et al., 2021), both of which act as oncogenes that promote proliferation, migration and invasion of tumour cells and inhibit apoptosis; in addition, circCSPP1 was found to promote liver metastasis and reduce Adriamycin sensitivity in CRC through different pathways. circFBXW7 (Lu et al., 2019; Xu et al., 2021) and circFNDC3B (Pan et al., 2020; Zeng et al., 2020) function as tumour suppressor genes and both inhibit cancer cell proliferation, migration and invasion. Moreover, circ-FBXW7 can improve chemoresistance to oxaliplatin in CRC by binding to miR-128-3p, and circFNDC3B can promote angiogenesis and liver metastasis.

Exosomes are nanoscale spherical lipid bilayer vesicles secreted by cells containing DNA, RNA, proteins, lipids, and other biologically active substances (Zhang et al., 2020c). Both normal and tumour cells can produce exosomes, which are widely distributed in various biological fluids, such as saliva, blood, urine, and ascites bile (Zhang et al., 2020c). Exosomes produced by tumour cells not only play an important role in tumour growth, metastasis and immunomodulation (Poggio et al., 2019; Sanderson et al., 2019), but also monitor disease progression and serve as diagnostic markers of the disease (Zhang et al., 2020c). Recent studies have shown that circRNAs are enriched and stable in exosomes, which confers the potential of exosomal circRNAs as biomarkers and new therapeutic targets for tumours (Wang et al., 2019b). In this study, we identified 32 exosomal circRNAs, most of which were found in serum or plasma. Maybe for the reason that blood samples from cancer patients are easier to be obtained. In contrast, saliva, urine and other body fluid samples are rarely collected in research. We suggest that future researchers should not only collect cancer tissues, but also try to collect serum, urine and saliva to help discover more exosomal circRNA forms in CRC.

In addition to the research on exosomal circRNAs to be improved, there are still some deficiencies in the research of circRNAs in colorectal cancer. For example, the expression levels of some circRNAs are inconsistent as reported in different articles. For controversial circRNAs, additional sample sizes for studies are needed to obtain reliable expression data. Alternatively, some articles do not go far enough into the study of circRNAs and lack exploration of specific mechanisms of action and pathways. Correspondingly, research on the mechanisms and pathways associated with circRNAs in tumours needs to be increased. Moreover, the current study just demonstrated that circRNAs proved to be highly stable in serum. For example, Li et al. found that human serum exosomes contain large amounts of intact and stable circRNA. They demonstrated that circRNA is resistant to digestion by RNase R exonuclease and incubation of serum at room temperature for up to 24 h showed little effect on circRNA levels (Li et al., 2015). When is diagnostic circRNA produced in the blood during cancer evolution, and how does the concentration of circRNA change after it is produced? How long does it last? These questions are worth exploring in the future.

In the last decade we have witnessed a revolution in single-cell transcriptomics (Aldridge and Teichmann, 2020). Single-cell sequencing is a technical means of separating cell populations in tissues or body fluids into individual cells and analyzing their genetic material by high-throughput sequencing (Lei et al., 2021). Unlike traditional sequencing methods, single-cell sequencing provides unbiased, high-throughput and high-resolution transcriptome analysis of individual cells, thereby revealing the gene structure and gene expression status of individual cells, reflecting the heterogeneity between cells and categorizing them for personalized analysis (Natarajan et al., 2019). As cellular heterogeneity is prevalent in tumors, understanding the heterogeneity of tumour cells has important implications for diagnosis, clarification of the nature of the tumour and targeted treatment (Chen et al., 2020b). Therefore, this technology can be applied to detect molecular phenotypic typing of circulating tumour cells, detect early tumour cells, monitor intra-tumour heterogeneity and guide targeted precision therapy (Rantalainen, 2018; Winterhoff et al., 2019). In 2022, Wu et al. explored the cellular landscape of circRNAs using full-length single-cell RNA sequencing to investigate the expression of circRNAs in neurons in the human brain and circRNAs in breast cancer cells, extending knowledge of circRNA expression to the single-cell level (Fan et al., 2015). However, single-cell analysis of circRNA in colorectal cancer is currently lacking, and we believe this is a highly promising area for future research.

To our knowledge, this study is the first systematic analysis focusing on CRC circRNA as a diagnostic, therapeutic and prognostic marker. Our literature analysis is relatively objective and comprehensive, recording the marker types, research methods, mechanism of action, function, expression, sample size and other indicators of these circRNAs. However, this study has some limitations. First, the literature search deadline was 4 December 2021, and subsequent studies were not included. Second, the search database was limited to PubMed. Third, only articles written in English were included in our analysis, which may introduce some linguistic bias. Fourth, we only recorded circRNAs associated with diagnostic, therapeutic, and prognostic markers. Finally, due to the large number of circRNAs, we only focused on the circRNAs that were repeatedly reported. Therefore, the circRNAs included in our analysis may not fully reflect all circRNA studies. However, our analysis covers the vast majority of circRNAs as biomarkers in CRC, which is enough to illustrate the basic situation of the research on circRNAs as CRC markers in recent years.

## 5 Conclusion

In this study, we summarized and analyzed the expression patterns, biological functions and clinical significance of circRNAs and related miRNAs, host and target genes in colorectal cancer. Exosomal circRNAs may be the most promising biomarkers in the future due to their specific mode of existence. In addition, we have summarised circRNAs associated with metastasis, drug resistance and radioresistance. Finally, we analyzed the current status and shortcomings of circRNA research on colorectal cancer according to the summary and make some suggestions.

## Data Availability

The original contributions presented in the study are included in the article/Supplementary material, further inquiries can be directed to the corresponding author.
